# The social and emotional burden of Dravet syndrome on Spanish caregivers

**DOI:** 10.1016/j.heliyon.2024.e34771

**Published:** 2024-07-17

**Authors:** Naiara Sánchez Marco, Simona Giorgi, José Ángel Aibar

**Affiliations:** Dravet Syndrome Foundation Spain, Madrid, Spain, C/ Toledo, 46, 1°, 28005, Madrid, Spain

**Keywords:** Dravet syndrome, Family unit, Social impact, Emotional impact, Caregivers, Parents

## Abstract

**Background:**

Dravet syndrome (DS) is a rare developmental and epileptic encephalopathy that presents with frequent and prolonged seizures resistant to treatment as well as cognitive problems such as behavioral and developmental delays. However, there is a lack of scientific literature on the impact of this condition on caregivers and the family unit.

**Objectives:**

To find out the social and emotional impact of DS on the family unit, to provide a comprehensive understanding of the disease's effects on both the family and caregivers.

**Materials and methods:**

A tailored online survey was administered to Spanish DS families, collecting data on the employment, financial, emotional, and social status of patients and caregivers.

**Results:**

A total of 112 Spanish caregivers participated in the study. The mean age of the 112 parents was 46.61 years, and 77.68 % of them were mothers. The majority of caregivers had to quit their jobs or reduce their working hours to take care of their child with DS, being the most of them mothers. Most of the caregivers felt that they were not well-informed by healthcare professionals (HCPs) and the Spanish National Health System (NHS). Despite access to resources, families often face financial strain and challenges in obtaining sufficient support, highlighting the need for enhanced social, economic, and psychological backing. In addition, both sentimental and social relationships were negatively impacted in the vast majority of respondents.

**Conclusions:**

The study advocates for policy reforms, integrated social services, community programs, and multidisciplinary efforts to improve the quality of life and social integration for those affected by DS.

## Highlights

• Mothers of people with Dravet syndrome (DS) tend to be the primary caregivers.
• Caregivers felt misinformed about the disease by the Spanish NHS and HCPs.
• Caring for a DS patient poses an emotional burden and affects the family situation.
• Caregivers' career, social relations and leisure activities are altered due to DS.
• Accessing public resources in Spain is time-consuming and they are usually insufficient.

## 1 Introduction

Dravet syndrome (DS) is a rare and severe developmental and epileptic encephalopathy that begins in childhood, usually before the first year of life. About 80 % of cases of DS are caused by a mutation in the *SCN1A* gene, which encodes for the sodium voltage-gated channel Na_v_1.1 [[1], [2], [3]]. The incidence of DS ranges from 1/15,700 to 1/40,000. Currently, in Spain, the prevalence is around 348–540 patients with DS [4,5].

DS is initially characterized by the presence of prolonged, generalized tonic-clonic or hemiclonic seizures, often triggered by fever or vaccination. However, other seizure types may also occur at onset [6,7]. DS patients experience frequent episodes of *status epilepticus* and present an increased risk of sudden unexpected death in epilepsy (SUDEP). Furthermore, seizures are resistant to common antiseizure medication [[8], [9], [10], [11]]. In adolescence and adulthood, seizures tend to decrease in frequency, but they rarely disappear. DS patients also present other associated comorbidities, such as intellectual disability, motor problems, behavioral problems, language impairment, and recurrent respiratory infections, which impact patients' and their caregivers’ quality of life (QoL) [7,[12], [13], [14], [15], [16]]. DS imposes a high economic burden on families. Different studies conducted in Spanish families showed that a significant percentage, ranging from 49 % to 72 %, did not receive any financial support to cover the costs of therapies for comorbidities associated with DS [17,18].

At the social level, people with epilepsy (PWE) often face challenges in developing community activities, socializing with others, and even carrying out activities with their peers in certain age groups. This considerably increases the sense of isolation in PWE and their families [5,13]. Several studies have also shown that parents and caregivers of people with DS experience significant physical and emotional impact. Caregiving can hinder the performance of relatively simple tasks and increase levels of anxiety and depression. Among others, studies have identified sleep deprivation, deterioration of social relationships and financial burden as the most significant factors that affect caregivers' lives [[19], [20], [21], [22]].

This situation worsened with the COVID-19 pandemic, during which 76 %–80 % of caregivers presented with new symptoms of depression and anxiety. This is believed to be related to a worsening in the behavior of the person with DS during confinement [23,24]. Additionally, more than 50 % of parents reported that having a child with DS has a permanent disruptive effect on their social life and relationships with family members and friends almost permanently [25,26]. In the professional sphere, 80 % of parents stated that caring for their DS child had an impact on their career decisions [27]. In terms of the healthcare system for persons with DS in Spain, it was shown that there is no standardized follow-up for DS patients beyond the age of 18, and mortality rates are uncertain. Diagnosis confirmation can take 9–15 months, and genetic testing availability is uneven. Treatment options include valproic acid, clobazam, stiripentol, and topiramate, but poor efficacy and safety often lead to treatment switches [28]. Previous studies highlighted the need for epidemiological studies, consensus criteria, easy access to genetic testing, treatment options, training, and research into quality of life aspects.

Thus, we aimed to delve into the impact of DS on the social and emotional component of caregivers’ lives.

## 2 Methods

### 2.1 Study design

To further investigate the social and emotional situation of Spanish caregivers of patients with DS, a tailored online survey was developed and administered to families of patients with DS living in Spain.

### 2.2 Participants and setting

Participants to this study were recruited via both the Dravet Syndrome Foundation Spain's newsletter and its social networks, meaning that we recruited participants that were either registered or not with the Foundation. The inclusion criteria required that participants be caregivers of a patient with Dravet Syndrome in Spain and be over 18 years old. Exclusion criteria included not meeting the inclusion criteria or not signing the informed consent form.

### 2.3 Research tool

After a thorough literature review, a survey was designed in order to capture the social and emotional burden of DS on the family unit. The survey was designed with user-friendly questions and it is for the most part close-ended. The main themes addressed in the survey were the caregivers’ employment status, their knowledge about DS, their family situation, their social relations and leisure time, and the information and resources available and which of them they received. The survey was designed for online completion by families and it was made available through an easily accessible tool on the www.dravetfoundation.eu platform.

### 2.4 Data collection

The survey was sent on September 13, 2021, and it remained open until October 12, 2021. During this period, reminders were sent to families through Dravet Syndrome Foundation's newsletter and posts were published on social media. Data were stored in Dravet Syndrome Foundation webpage which is hosted in Microsoft Azure cloud platform. Azure has all relevant information security and cloud certifications, including ISO 27001, ISO 27701 and CSA STAR and encrypts data using FIPS 140-2 compliant 256 AES encryption for storage accounts and virtual machine disks.

### Ethical approval

The study was conducted in accordance with the Helsinki Declaration and approved by the Ethics Committee for Research at Universidad Rey Juan Carlos in Madrid, Spain (registration number 0206202115721). Before starting the survey, participants were given the study information sheet and the informed consent form, which was electronically signed before participation in the study. Personal data were dissociated from the results in compliance with the EU General Data Protection Regulation (GDPR).

### 2.5 Data analysis

Data analyses and graphical representations were carried out using Microsoft Excel and GraphPad 8.0.1. Categorical variables were represented as frequencies (bar graphs and pie chart) by counting the number of occurrences for each variable. Continuous variables were represented as scatter dot plot. Categorical variables were compared with the Fisher exact test with a significance level of 5 % or the Chi-square test with a significance level of 5 %. Correlations between variables were calculated by regression analysis and *R*^2^. Continuous variables were analyzed by Shapiro-Wilk normality test and compared by Kruskal-Wallis.

## 3 Results

### 3.1 Demographic data

One hundred and twelve parents completed the survey, 87 of them were mothers (77.68 %). The mean age of the participants was 46.61 years (±8.27, range 28–71 years) (Table S1). According to the survey, 91.07 % (102/112) of the participants were primary caregivers and, mothers were more likely to be primary caregivers than fathers (p = 0.0079). Among the 8.93 % (10/112) who were not the primary caregivers, 6 were fathers and 4 were mothers (Table 1). The age of individuals with DS whose families participated in the study varied widely, with a mean of 13.30 years (±9.26, range 0.5–41 years) (Table S1). The majority of patients with DS (56.25 % [63/112]) received a correct diagnosis between 1 and 4 years of age.

**Table 1:** Gender distribution among caregivers.

	Respondents (N; %)	Primary caregivers (N; %)	Not primary caregivers (N; %)
Mother	87; 77.68	83; 95.40	4; 4.59
Father	25; 22.32	19; 76.00	6; 24.00

**Fig. 1:**
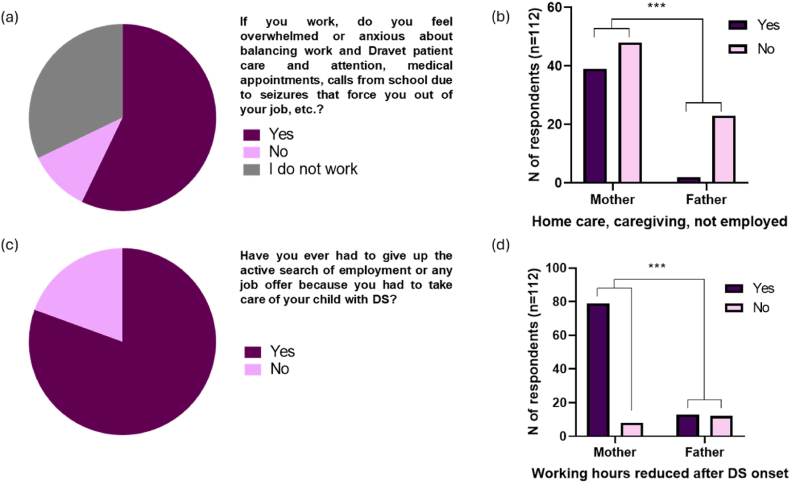
Employment situation (a) Feeling overwhelmed or anxious about reconciling work with care and attention of the person with Dravet syndrome (n = 112); (b) Percentage of mothers and fathers involved in home care, caregiving or currently unemployed (n = 112) (Fisher exact test ***p = 0.0007); (c) Obligation to give up the active search for employment or any job offer (n = 36); (d) Percentage of mothers and fathers who did not reduce their working hours due to DS (n = 112) (Fisher exact test ***p = 0.0002). 18.

The residence of these families was widely spread throughout Spain, with Madrid being the region with the highest number of families (21.43 %, [24/112]), followed by Andalusia (19.64 % [22/112]) (Table S2). Most participants were married (68.75 % [77/112]) and half of the families consisted of 4 members (Fig. 1). As for the highest level of education attained, most of them (41.96 %, [47/112]) were high school graduates or pursued vocational training at intermediate or higher levels, and 30.36 % of respondents (34/112) graduated with a bachelor's degree (Table S3). Regarding higher education levels (college or post-college), 56.00 % (14/25) were fathers, while 40.23 % (35/87) were mothers. No correlation was found between these two variables (p = 0.1772).

The employment status of the parents participating in the survey was divided into full-time employed (24.11 %, [27/112]), home or family caregiver (19.64 %, [22/112]), part-time employed (13.39 %, [15/112]), unemployed (13.39 %, [15/112]), self-employed (12.50 %, [14/112]), retired (5.36 %, [6/112]), employer with employees (2.68 %, [3/112]) and other (8.93 %, [10/112]) (Table S3). A great amount of the respondents who did work (84.21 % [64/76]) felt overwhelmed and anxious about balancing and caring for the person with DS (Fig. 1a). As shown in Fig. 1b, mothers were more involved in home care, caregiving for the person with DS, or they were not employed, with 44.83 % (39/87) of mothers compared to only 8.00 % (2/25) of fathers (p = 0.0007). Respondents were also asked whether they had to give up the active search for employment or any job offer and 80.56 % (29/36) stated that they had to (Fig. 1c). Some participants even expressed that they (14.29 %, [16/112]) or their partner, spouse, or other family member (17.86 %, [20/112]) had been forced to work multiple jobs. The majority of caregivers (73.21 %, [82/112]) had to leave work or reduce their working hours to care for their child with DS (Fig. 1d). Among the caregivers who were able to maintain their regular working hours, the majority were fathers (48.00 % [12/25]), in contrast to only 9.20 % (8/87) who were mothers (p < 0.0001).

### 3.2 Knowledge about Dravet syndrome

**Fig. 2:**
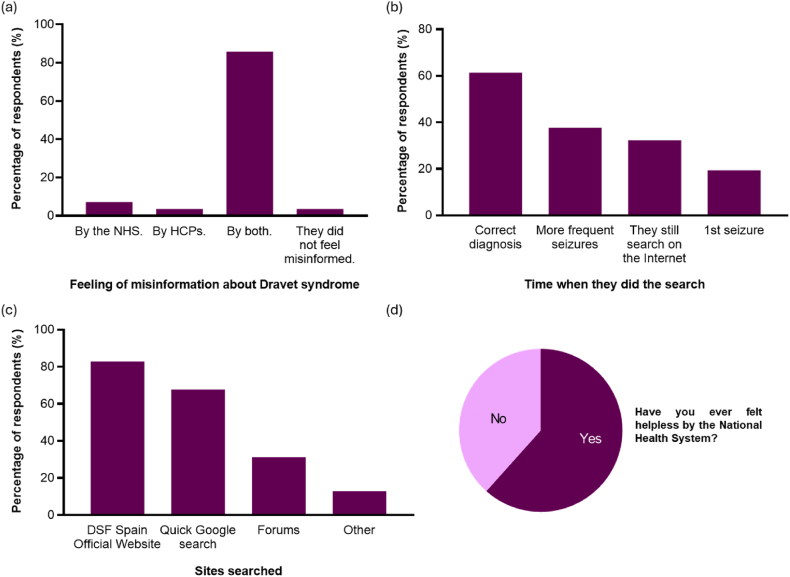
Knowledge about DS (a) Feeling of misinformation about Dravet syndrome (n = 112); (b) Time at which respondents turned to Internet searches (n = 93); (d) Sites searched (n = 93); (d) Feeling of being helpless from the NHS (n = 112). 19.

As shown in Fig. 2a, 85.71 % of respondents (96/112) stated that they felt misinformed both by the National Health System (NHS) and by healthcare professionals (HCPs). The misinformation sentiment from the 10.13039/100030827NHS encompasses mainly logistical issues, information resources, and lack of support in unconventional therapies, among others.

In general, 83.93 % (94/112) replaced professional information with Internet searches. As shown in Fig. 2b, most caregivers (61.29 %, [57/93]) reported having done their searches upon diagnosis, while the minority (19.35 %, [18/93]) did so after the first seizure. Topping the list of websites visited by caregivers is the Dravet Syndrome Foundation Spain official website with 82.80 % (77/93) of the searches, followed by quick Google searches (67.74 % [63/93]) (Fig. 2c).

Furthermore, 94.64 % of caregivers ([106/112]) stated that they had the feeling that they knew more or better about the condition than the HCPs who cared for their child, and 61.61 % (69/112) felt helpless by the NHS (Fig. 2d).

Parents were also asked how they felt when they received the DS diagnosis (Table 2). Many of them identified negative feelings related to sadness, despair, distress, sinking and concern (68.75 %, [77/112]). They also felt confusion, helplessness and uncertainty (30.36 %, [34/112]). However, some others (17.86 %, [20/112]) were relieved to finally have a correct diagnosis, mainly because they had been misdiagnosed many times before.

**Table 2:** Feeling upon receiving the correct diagnosis.

Feeling	Percentage of respondents (%)
Sadness, distress, concern, rage/anger, sinking, despair, etc.	68.75
Confusion, helplessness, fear, lack of information, uncertainty for the future, etc.	30.36
Relief, satisfaction, acceptance, being able to contact the Foundation, etc.	17.86
Frustration due to late diagnosis	2.68
Loneliness	2.68
Just a name	1.79
No definitive diagnosis yet	1.79
Denial	0.89
Lack of resources to cope	0.89

### 3.3 Family situation

**Fig. 3:**
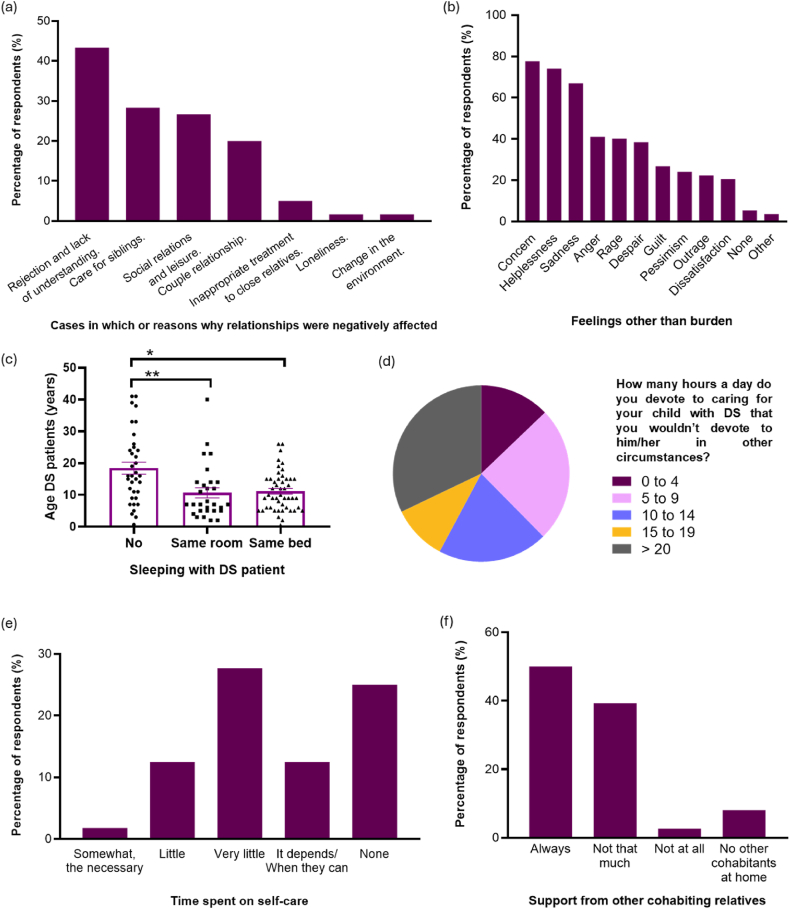
Family situation (a) Cases in which or reasons why the relationship with other family members has been negatively affected by having to take care of the person with DS (n = 60); (b) Feelings, other than burden, when coping with care and household responsibilities simultaneously (n = 112); (c) Caregivers sleeping with the person with DS in relation to the age of the patient. (Shapiro-Wilk normality test, Kruskal-Wallis **p = 0.0020; *p = 0.0114) (d) Hours per day spent on caregiving (n = 109); (d) Time that caregivers devote to self-care (n = 112); (d) Support that caregivers receive from other cohabiting relatives (n = 112). 20.

When asked about the family situation, 63.39 % (71/112) of caregivers stated that the disease had strengthened the family bond, and more than half of them reported that it had been strengthened much more than before. On the other hand, this situation changed families’ home routines 93.75 % of the time (105/112) in all aspects of daily life. However, some of them found benefits to the situation, such as shifting their priorities and minimizing other problems (16.07 %, [18/112]), being more patient and empathetic (15.18 %, [17/112]), or spending more time with their family or children (13.39 %, [15/112]) (Table 3). In contrast, 52.68 % of the respondents (59/112) reported negative impacts on their relationship with other family members due to different reasons, such as rejection and lack of understanding by the rest of the family or the care for siblings (Fig. 3a). Most respondents (66.96 %, [75/112]) also reported feeling burdened by the need to balance caring for the person with DS and household responsibilities. Furthermore, many caregivers reported feeling sad (66.96 %, [75/112]), helpless (74.11 %, [83/112]) or concerned (77.68 %, [87/112]), among other feelings, due to coping with both care and household responsibilities simultaneously (Fig. 3b). In 42.86 % of the cases (48/112) the person with DS slept in the same bed as their parents and 25.89 % (29/112) slept in the same room, showing how the intimacy of the couple may be impacted. As shown in Fig. 3c, caregivers tend to sleep in the same room or in the same bed less with older DS patients compared to younger ones (same room p = 0.0020; same bed p = 0.0114).

**Table 3:** Aspects of their life that have benefitted.

	Percentage of respondents (%)
No benefits	28.57
Values, life priorities, minimizing problems	16.07
Patience, comprehension, empathy, strength	15.18
Family feeling, time with family or children	13.39
Love, admiration, affection	12.50
Living day by day, the small details	9.82
Becoming a better person	8.04
Enjoying more of their child	6.25
Watching them grow up	3.57
Do not know	3.57
Learning a lot from the person with DS	1.79
Feeling special	1.79
Satisfaction for what has been achieved	1.79
Meeting nice people	1.79
Feeling of plenitude	1.79
Couple relationship	0.89
Going out more	0.89
Joining Dravet parent associations	0.89
Peace	0.89

Regarding the amount of time spent caring for the child, most caregivers had very little (27.68 %, [31/112]) or no time (25.00 %, [28/112]) for self-care due to the hours spent caring for their child (Fig. 3d and e). In this regard, no correlations were found between the age of the person with DS and the amount of time spent daily on caregiving. Moreover, although half of the respondents (50.00 %, [56/112]) received support from other cohabiting relatives with their needs, many others (39.29 %, [44/112]) did not receive as much support as they would have liked to (Fig. 3f). Thus, many of them felt highly overburdened and have considered delegating some responsibilities to continue caring for the person with DS (65.18 %, [73/112]), of which 36.99 % (27/73) already have external support. Out of the 27 caregivers who rely on external support, 7 (25.93 %) reported feeling negatively about themselves due to it, all of them mothers, with 4 (57.14 %) experiencing these feelings on a daily basis. Additionally, 20.54 % of respondents (23/112) indicated that they do not allow themselves to feel bad or express negative emotions in front of the person with DS.

### 3.4 Social relations and leisure

**Fig. 4:**
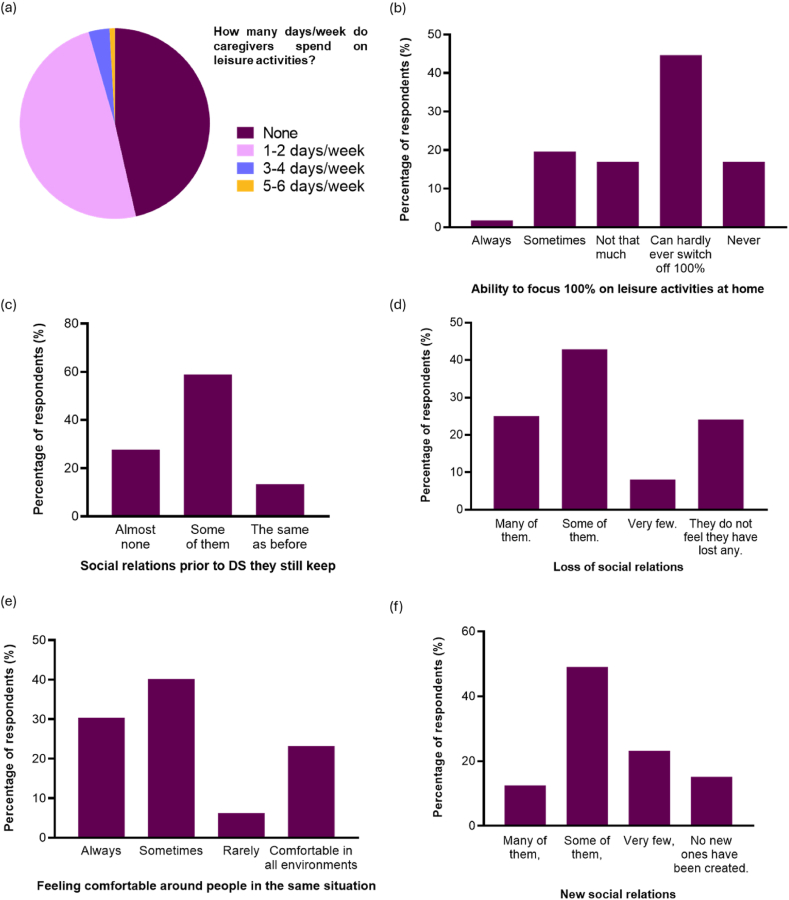
Social relations and leisure. (a) Days per week devoted to leisure activities (n = 112) (b)Leisure activities at home (n = 112); (c) Modified social relations (n = 112); (d) Loss of social relations (n = 112); (e) Feeling more comfortable with people in the same situation (n = 112); (f) New social relations (n = 112). 21.

Most caregivers spent either 1 or 2 days (49.11 %, [55/112]) or no days (46.43 %, [52/112]) of the week on leisure activities (Fig. 4a). Regarding the leisure activities they do at home, many parents (44.64 %, [50/112]) identified that they can hardly ever be 100 % attentive to the activity, compared to a very small percentage (1.79 %, [2/112]) who always do such activities without any problems (Fig. 4b). On the other hand, 42.86 % of the respondents (48/112) reported not engaging or participating in any leisure activities outside of their home, either alone or with friends or family members for whom they are not in charge. Only 52.68 % (59/112) engaged in specific leisure activities without the person with DS.

In addition to this, 88.39 % (99/112) of parents had to seek out more suitable environments to spend time with the person with DS. This is due to the fact that 92.86 % (104/112) do not believe there are enough adapted leisure spaces in which they can safely carry out such activities with their child with DS, such as going to the park or to the cinema. Thus, in 58.93 % of the cases (66/112), their social relationships were modified to some extent, as they only maintained their closest relationships. However, the time spent daily on caregiving did not correlate with the loss of social relationships (Chi-square test p = 0.2083). Furthermore, there was general agreement among caregivers that being with people in the same situation makes them feel understood and comprehended, they feel comfortable as they do not feel judged, and allows them to help each other since they have much in common. Thus, caring for a person with DS also resulted in some new social relationships in 49.11 % of the respondents (55/112) (Fig. 4c–f).

### 3.5 Information and resources

**Fig. 5:**
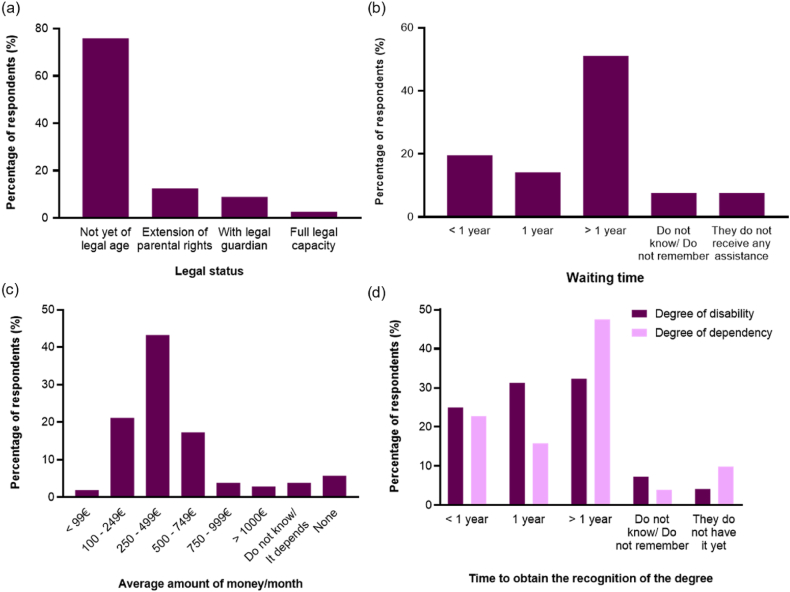
Information and resources (a) Legal status of the person with DS (n = 112); (b) Waiting time to receive any public assistance (n = 98); (c) Average amount of money spent monthly on care, treatment, therapies, and therapeutic items not covered by public administrations (n = 104); (d) Time to obtain the recognition of the degree of disability (n = 96) and dependency (n = 101). 22.

Finally, we examined the legal status of individuals with DS and their access to social resources. Only 24.11 % of persons with DS (27/112) were of legal age and around half of their caregivers (51.85 %, [14/27]) were granted the legal extension of parental rights (Fig. 5a). Also, more than half (53.57 %, [60/112]) of the caregivers reported to know public or private social resources that could assist them, such as support at work or at school, family respites, and recognition of the degree of dependency and disability, among others. Indeed, 89.29 % of families (100/112) had access to some type of social resources and 90.00 % of those who had access (90/100) could benefit from them, being financial support and rehabilitation services the most accessed resources (60.71 %, [68/112]; and 35.71 %, [40/112]; respectively). The institutions providing the most social support were the regional social services (43.75 %, [49/112]) and the city hall or municipal services (33.93 %, [38/112]). The majority of respondents stated that the most important things for them when applying for public aid or support are the ease of access to information and requirements (37.84 %, [42/112]) and a resolution that is beneficial to family (41.44 %, [46/112]) (Table 4).

**Table 4:** Access to resources and institutions providing the assistance.

	Percentage of respondents (%)
Benefit or access to resources	
Financial support	60.71
Rehabilitation services (Occupational Therapy, Physiotherapy, Speech Therapy …)	35.71
Social welfare services	14.29
They do not benefit from or have access to any resources.	10.71
They do not benefit from any	8.93
Other	6.25
Psychological assistance services	5.36
Personal care assistant	3.57
Help with household chores	1.79
Telephone support	0.89
***Institution providing social welfare services***	
Regional social services	43.75
City Hall or municipal services	33.93
Non-profit foundations and organizations	16.07
None	14.29
Regional healthcare system	12.50
Other	8.04
Provincial Council	4.46
***Important things when applying for public aid***	
That the resolution is beneficial to the family.	41.44
Easy access to the information and requirements.	37.84
Easy access to the resource.	19.82
Other	0.90

When asked whether they had easy access to public system assistance, 64.86 % of respondents (72/111) said it was not easy. Although the waiting time to receive any type of public support after requesting it, is very variable, most families had to wait for over a year to access it and 82.14 % of them (92/112) considered that they do not receive sufficient support from public administrations for the care of a person with DS. Most families (64.42 %, [67/104]) spend around 100–499€ monthly on care, treatment, therapies, and therapeutic items that are not covered by public administrations (Fig. 5b and c).

As for the procedure to obtain the recognition of degree of disability and dependency, the social or health services explained to most families how it works and the related support available (82.73 %, [91/110]). However, 43.15 % of caregivers (42/91) did not understand their explanation. As shown in Fig. 5d, it took more than a year to receive the recognition of disability or dependency degrees. For both the disability and the dependency degrees, the majority of families (67.27 %, [74/110]) were satisfied with the assessment received.

## 4 Discussion

### 4.1 Demographic data

This study aimed to assess the social and emotional impact of DS on caregivers and the family unit in Spain. Although the number of participants in the study was limited due to DS being a rare disease, parents of patients with DS showed great interest in participating in the study, considering the length of the survey and the short time the survey was open. This reflects their will to investigate the problems arising from DS at a social level and improve their situation through research. Our study shows that the majority of participants were mothers and that they are more likely to be the primary caregivers, which is consistent with previous studies showing that women typically assume this role for individuals with DS [25,29,30].

Data obtained in previous studies show the challenging employment situation faced by caregivers of individuals with DS [31,32]. The results of the present study provide further evidence in this regard, as the majority of the participants either left their job or reduced their working hours to cope with the situation at home. Besides this, those who continue to work often feel overwhelmed or anxious about balancing work with caregiving responsibilities.

### 4.2 Knowledge about Dravet syndrome

We also found that most caregivers felt misinformed about the disease by the NHS and HCPs, which led them to replace that missing information by searching on the Internet, especially on the Dravet Syndrome Foundation Spain official website. Interestingly, some caregivers reported having searched for scientific material on other countries’ websites to learn more about DS and its impact on the QoL of patients and families. This is consistent with the fact that studies on the impact of DS have been conducted mainly in other countries such as the USA, UK or Canada, among others [[33], [34], [35]], which highlights the importance of carrying out this type of studies in our country. Based on the study findings, HCPs are encouraged to adopt a more proactive role in educating families about DS, policymakers should consider the creation of targeted support programs that address the unique challenges of rare diseases, while patient advocacy groups can play a pivotal role in raising awareness and driving research initiatives that focus on the social and emotional aspects of DS caregiving.

### 4.3 Family situation

As shown by our results and previous studies, caring for a person with DS imposes an emotional burden on caregivers [18,21,32,35]. In addition, more than half of the respondents reported that other family members refrain from participating in the care of the person with DS due to the challenges it presents. Thus, these family members may reject the person with DS and their parents, and even create distance between themselves and family. A previous study from Domaradzki and Walkowiak reported that DS caregiving has an emotional impact and can worsen the relationship with other family members, in addition to a negative impact on the couple's relationship [36]. Similarly, Jensen et al. identified a decline in the family relationship as the disease progresses [37]. However, our study did not find any correlation between the patient's age and the decline in the family relationship. Our results show that most parents felt sad, helpless, or concerned about balancing caregiving with household responsibilities, and more than half of them stated that the relationship with other family members has been negatively affected by the situation. Moreover, the intimacy of the couple also seemed to be affected in many cases, partly due to the person with DS sharing a same bed or room with parents. It is also noteworthy that caregivers tend to share a sleeping space more frequently with younger patients as opposed to adults.

### 4.4 Social relations and leisure

In a large proportion of cases, social relationships were also affected and modified because of DS. Many parents reported that having a child with DS allowed them to form new friendships, but they also lost some of their friendships prior to the disease onset. Additionally, we identified that caregivers experience a significant lack of time for themselves as well as for leisure activities, both at home and outside. This, added to the lack of sufficient adapted leisure spaces, makes it difficult to develop a fulfilling social life. These results are consistent with findings shown in previous studies [21,36] and show that DS has a negative impact not only on the family nucleus, but also on the social component of caregivers’ life.

### 4.5 Information and resources

Another key result from our study is that while most parents had access and could benefit from public resources, including financial and rehabilitation assistance, accessing them was not easy, and the process took several years. Additionally, the great majority of caregivers felt that the support received from public administrations for the care of a person with DS was insufficient. Compared to common chronic conditions, rare diseases pose a tremendous collective cost burden to most countries, although the lack of a standardized approach to calculating such data makes direct comparison challenging. Studies have shown that, in many European countries, this burden can exceed 50,000€ per patient for many rare diseases, some of them reaching up to or exceeding 200,000€ [38]. While previous works acknowledge the significant socioeconomic burden of DS [[39], [40], [41]], there is a lack of clear characterization and quantification as many studies rely on self-reported data and do not incorporate economic models [39].Future research should aim to explore the economic impact of DS on families, the effectiveness of different support services, and the development of interventions that can improve the social integration of DS patients.

### 4.6 Limitations of the study

As already mentioned, the number of participants was limited to 112 caregivers, predominantly mothers, which may not capture the full spectrum of DS caregiver experiences in Spain and include a gender bias to results. The length of the survey and the short time it was published may have also been determinants of the participation in the study. Another limitation is that the data collected were self-reported and subject to recall bias and social desirability bias, which may affect the accuracy of some of the responses. Also, the survey was only conducted in Spain, so the findings of this study are context-specific to Spain and may not be generalizable to other countries due to cultural, social, and healthcare system differences. However, our goal was to assess the emotional, social and economic impact of DS in the Spanish context. In addition, the tool was not a validated scale, as there are no specific scales for assessing the social and emotional burden on families of individuals with DS. As for the type of study conducted, this is a cross-sectional study that assesses the emotional and social situation of caregivers as well as the family situation at the time of the survey. To capture the evolving needs of DS families, it would be useful to conduct longitudinal studies providing valuable insights into the long-term effects of DS on caregivers and the effectiveness of support services. This approach would also allow for the identification of critical periods where intervention could have the most significant impact on improving the quality of life for both patients and caregivers.

### 4.7 Conclusions

Dravet syndrome, as well as other rare diseases, do not only impact directly patients' lives, but also place an indirect burden on their families’ social life and emotional state. In most cases, social, family and couple relations were affected, and many caregivers had to modify or give up their professional career. Respondents felt that everything adapts and revolves around their child with DS, that leisure and social relations decreased, or that everything must be scheduled. In addition to this, they generally had to give up their work/life balance, their personal life, or any activity other than caring for the person with DS. Furthermore, many families know and have access to financial or rehabilitation resources, but these are usually not sufficient to meet the needs of individuals and families with DS, resulting in many of them still having to spend a big amount of money on care and treatment. Along with this, access to these resources is by no means easy and it usually takes several years. Further research from a social point of view is needed in order to understand how to overcome these limitations, finally being able to support families of DS patients from a social, economic and psychological perspective.

The findings of this study underscore the need for policy reforms that specifically address the challenges faced not only by individuals with DS, but also by their families. Policies that facilitate access to comprehensive care, provide financial assistance, and promote educational programs for HCPs can significantly alleviate the burden on these families. Moreover, integrating social work services into the healthcare system could provide much-needed support in navigating the complex landscape of care for rare diseases, including DS. Also, strategies to improve the social integration of DS patients and their families should be a priority. Community-based programs, inclusive activities, and the creation of adapted leisure spaces can foster a sense of belonging and reduce the isolation often experienced by DS families. For these reasons, it is encouraged that future projects involve PAGs, clinicians, and social workers collaborating to develop tailored leisure activities, expand the spaces available for such activities, increase the number of daycare centers, and enhance public policies. These policies should aim to support families both emotionally and economically, ultimately improving the social well-being of patients and their families.

In addition, our data highlight the importance of the involvement of patients advocacy groups, which help informing families and may support them addressing the different conflicts identified. We urge considering our recommendations to improve our collective ability to capture and analyze data on DS and rare diseases and, ultimately, to improve the QoL of families affected.

In conclusion, a collaborative effort between researchers, clinicians, social workers, and families is essential to develop a multidisciplinary approach to managing DS. Such collaboration can lead to the creation of holistic support services that address not only the medical needs but also the social and emotional well-being of individuals and families with DS.

## Data availability statement

Data associated with this study is included either in the article or in supplementary material, or is referenced in the article.

## Funding sources

This study has been funded by the 10.13039/100009712Dravet Syndrome Foundation Spain.

## Ethical publication statement

We confirm that we have read the Journal's position on issues involved in ethical publication and affirm that this report is consistent with those guidelines.

## CRediT authorship contribution statement

**Naiara Sánchez Marco:** Writing – review & editing, Writing – original draft, Visualization, Methodology, Investigation, Formal analysis, Data curation. **Simona Giorgi:** Writing – review & editing, Writing – original draft, Visualization, Validation, Supervision, Methodology, Investigation, Data curation. **José Ángel Aibar:** Writing – review & editing, Writing – original draft, Supervision, Project administration, Funding acquisition.

## Declaration of competing interest

JAA is president and SG is Scientific Director of the Dravet Syndrome Foundation Spain (DSF). They and/or the DSF have received grants and/or financial support from GW Pharma, Zogenix, Ovid Therapeutics, Encoded Therapeutics, Biocodex, Praxis, Stoke, Takeda, UCB, Epygenix, Jazz Pharmaceuticals and StrideBio to help carry out some of the DSF's foundational activities or provide consulting services. The honoraria have always been donated directly or indirectly to the DSF.

NS has no conflicts of interest.
