# Supplementary material for: The social and emotional burden of Dravet syndrome on Spanish caregivers
Source: Heliyon. 2024 Jul 17;10(14):e34771. doi: 10.1016/j.heliyon.2024.e34771 (PMC11324968; doi:10.1016/j.heliyon.2024.e34771)
Supplement: Multimedia component 1 [file mmc1.docx]

**SUPPLEMENTARY MATERIAL**

**SUPPLEMENTARY TABLES WITH LEGENDS**

**Supplementary table 1.** Age of caregivers and DS patients

|  | **Range** | **Mean** |
| --- | --- | --- |
| **Age caregivers (years)** | 28-71 | 46.61 |
| **Age DS patients (years)** | 0.5-41 | 13.29 |

**Supplementary table 2.** Region and province of residence of the respondents.

| **Region** | **Province** | **Number of respondents** |
| --- | --- | --- |
| ***Andalucía*** | Almería | 1 |
|  | Cádiz | 3 |
|  | Córdoba | 2 |
|  | Huelva | 1 |
|  | Jaén | 1 |
|  | Málaga | 6 |
|  | Sevilla | 8 |
| ***Aragón*** | Zaragoza | 4 |
| ***Asturias*** | Asturias | 1 |
| ***Castilla La Mancha*** | Albacete | 1 |
|  | Ciudad Real | 1 |
|  | Toledo | 1 |
| ***Castilla y León*** | Burgos | 2 |
|  | León | 1 |
|  | Soria | 1 |
|  | Valladolid | 4 |
| ***Cataluña*** | Barcelona | 9 |
|  | Lleida | 3 |
|  | Tarragona | 1 |
| ***Comunidad Valenciana*** | Alicante | 5 |
|  | Castellón | 1 |
|  | Valencia | 7 |
| ***Extremadura*** | Cáceres | 4 |
| ***Galicia*** | Coruña | 2 |
|  | Pontevedra | 4 |
| ***Islas Baleares*** | Baleares | 2 |
| ***Islas Canarias*** | Las Palmas | 1 |
| ***La Rioja*** | La Rioja | 3 |
| ***Madrid*** | Madrid | 24 |
| ***Murcia*** | Murcia | 3 |
| ***Navarra*** | Navarra | 2 |
| ***País Vasco*** | Gipuzkoa | 2 |
|  | Vizcaya | 1 |

**Supplementary table 3.** Information on education and employment status.

|  | **Percentage of respondents (%)** |
| --- | --- |
| ***Highest level of education attained*** |  |
| High school | 14.29 |
| Baccalaureate, vocational training at intermediate or higher levels | 41.96 |
| Graduated with a bachelor’s degree | 30.36 |
| Postgraduate studies: master’s degree, doctorate… | 13.39 |
| ***Employment status of the parents/caregivers*** |  |
| Full-time employed | 24.11 |
| Home or family caregiver | 19.64 |
| Part-time employed | 13.39 |
| Unemployed | 13.39 |
| Self-employed | 12.50 |
| Other | 8.93 |
| Retired | 5.36 |
| Employer with employees | 2.68 |

**SUPPLEMENTARY FIGURES**

**SUPPLEMENTARY FIGURE 1. Demographic data**


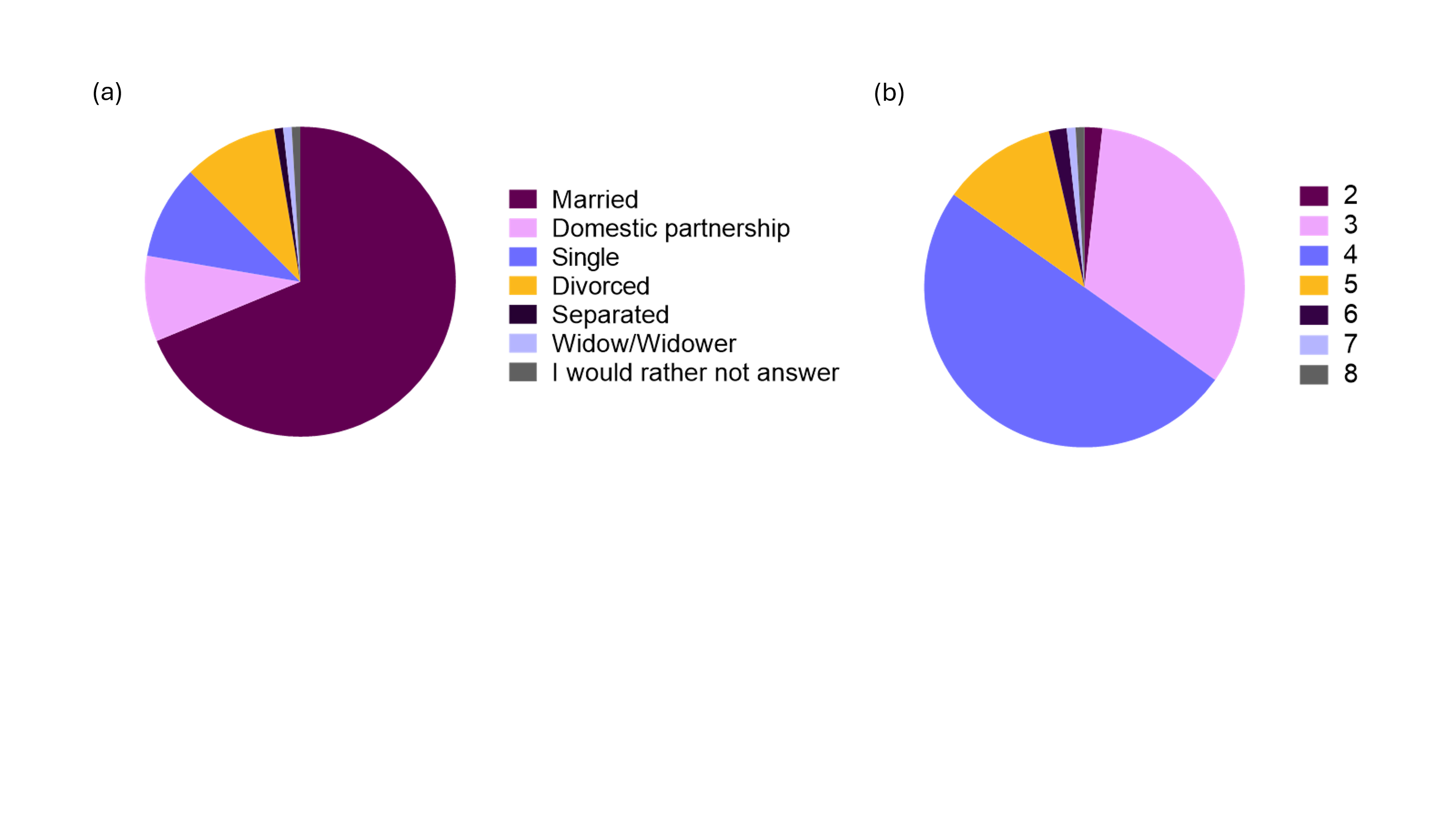

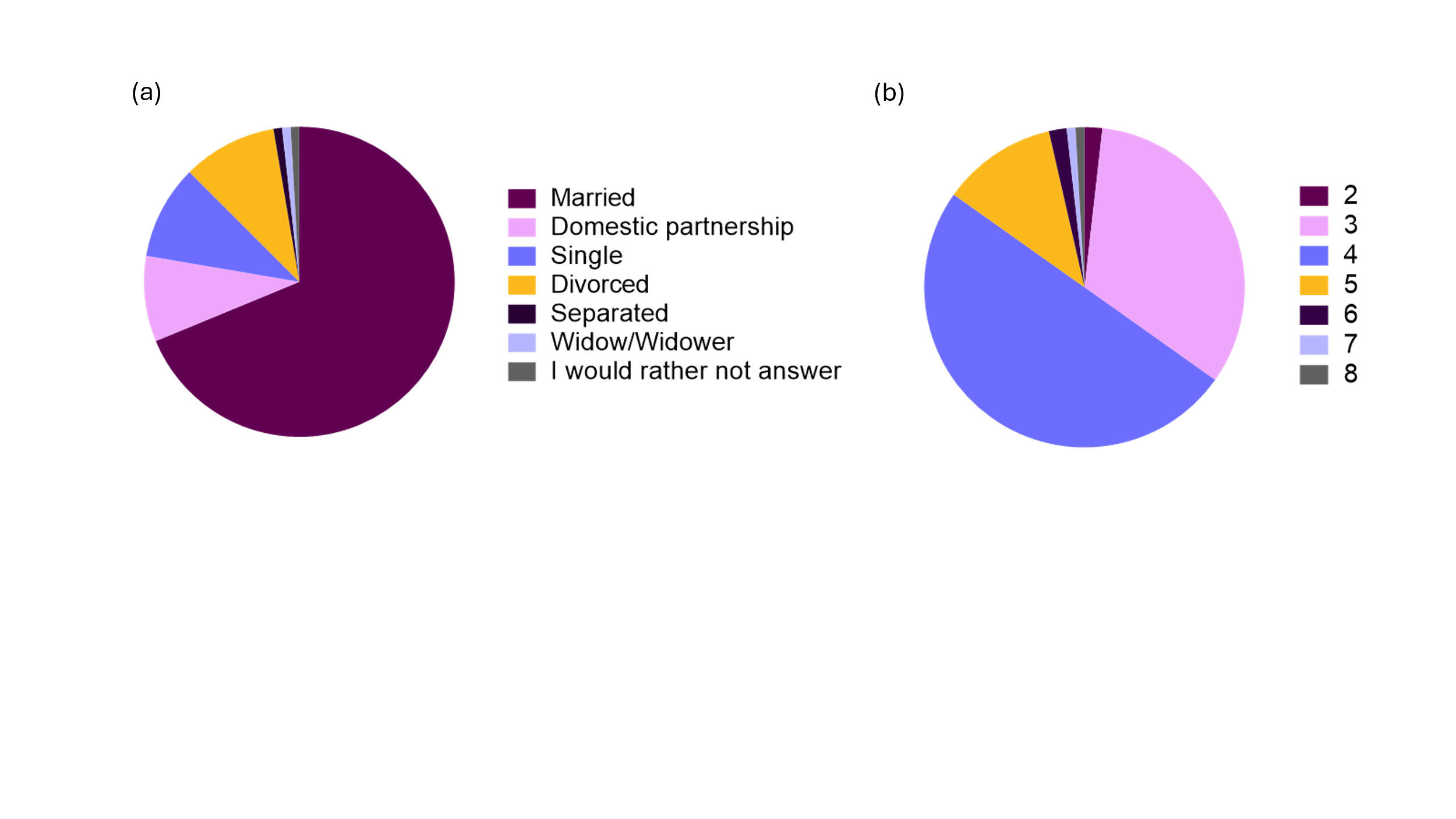


**Supplementary figure 1.** Demographic data. (a) Marital status of the respondents (n=112); (b) Number of household members (n=112).

**SURVEY**

**Section 1**. Informed consent.

**Section 2**.

1. Indicate with numbers how many members make up the household unit, including yourself:
2. Family role/relationship/relationship with the person with Dravet syndrome.
   - Mother.
   - Father.
   - Sibling.
   - Grandparent.
   - Other:
3. Are you the primary caregiver?
   - Yes.
   - No.
4. Indicate who assumes the role of primary caregiver

**Section 3**.

1. What is the sexual identity with which you identify?
   - Woman.
   - Man.
   - None of the above.
   - I'd rather not answer.
2. What's your age?
   - 18-24.
   - 25-34.
   - 35-44.
   - 45-54.
   - 55-64.
   - 65-74.
   - More than 75.
3. Indicate your province of residence
4. What is your marital status?
   - Single.
   - Domestic partnership.
   - Married.
   - Widow/widower.
   - Separated.
   - Divorced.
5. Select the highest level of education attained.
   - No education.
   - ESO, EGB or equivalent.
   - Baccalaureate, Vocational Training in intermediate or higher grades.
   - Bachelor's degree, diploma or graduate.
   - Postgraduate university studies: master's degree, doctorate, etc.
6. What is your employment status?
   - Student.
   - Unemployed.
   - Taking care of the home or family
   - Self-employed.
   - Employer with employees.
   - Part-time employee.
   - Full-time employee.
   - Retired
   - Other
7. What is the age of the person with Dravet syndrome?

**Section 4**.

- - 1. Do you feel that there is a lot of misinformation about Dravet Syndrome by the Public Health System and the health professionals themselves?
       - Yes, by both.
       - Only by the Public Health System.
       - Only by healthcare personnel.
       - No, I think they know a lot about Dravet syndrome.
    2. Have you replaced the lack of information on the part of professionals by looking for it through the internet?
       - Yes.
       - No.
    3. When did you resort to looking up that information on the internet?
       - When the crises began to be more frequent and there was no explanation from the professionals of what was happening.
       - As soon as we were given the correct diagnosis.
       - To this day, I still consult the internet.
    4. If you have searched the internet for information about Dravet Syndrome, where did you search?
       - Forums.
       - Quick Google search.
       - Official website of the Dravet Syndrome Foundation.
       - Other:
    5. Have you ever felt that you are the one who knows more about Dravet syndrome than the health professional in front of you?
       - Yes.
       - No.
    6. Have you ever felt helpless by the National Healthcare System?
       - Yes.
       - No.
    7. At what age were you given the correct diagnosis?
    8. Briefly explain how you felt the first time you received a diagnosis.

**Section 5**.

1. Have you been forced to be or are you currently moonlighting in order to pay for the necessary care of the person with Dravet Syndrome and the rest of your dependents?
   - Yes.
   - No.
2. If you have a partner, spouse or other family member who can help you, has he/she been the one who has had to hold several jobs?
   - Yes.
   - No.
   - I don't have a partner, spouse, or other family member to help us.
3. Did you leave your job or reduce your working hours to take care of your child with Dravet syndrome?
   - Yes.
   - No, I continue with my working day without change.
   - No, I was not working when I had my child with Dravet syndrome.
4. If you work, do you feel overwhelmed or anxious about the reconciliation of work with the care and attention of the Dravet patient, medical visits, calls from school due to crises that force you to leave the job, etc.?
   - Yes.
   - No.
   - I don't work.
5. Did you actively give up looking for a job because you have to take care of your child with Dravet syndrome?

- Yes.
- No.

**Section 6**.

1. Do you feel that caring for a person with Dravet Syndrome has strengthened the family bond?
   - Yes.
   - No.
2. To what extent has it been strengthened?
   - Much more than before.
   - Something more than before.
   - Slightly reinforced.
3. Have your routines changed at home due to Dravet Syndrome?
   - Yes.
   - No.
4. With respect to the previous question, how have they been modified?
5. Do you think caring for your family member with Dravet syndrome negatively affects your relationship with other family members?
   - Yes.
   - No.
6. If the answer above was yes, please explain in which cases they have been adversely affected and why you think this has happened and with whom.
7. Are you overwhelmed by having to care for the person with Dravet syndrome while maintaining the rest of your responsibilities at home?
   - Yes.
   - No.
8. What other feelings would you describe in addition to overwhelm?
   - Sadness.
   - Impotence.
   - Anger.
   - Indignation.
   - Fault.
   - Rabies.
   - Worry.
   - Despair.
   - Pessimism.
   - Dissatisfaction.
   - Other:
   - None.
9. Generally speaking, do you feel like you don't have enough time for yourself?
   - Yes.
   - No.
10. Does the person with Dravet syndrome sleep in the same bed or room as either parent?
    - Yes, in the same bed.
    - Yes, in the same room.
    - No.
11. Do you think this has negatively affected the couple's intimacy?
    - Yes.
    - No.
    - I'd rather not answer.
12. On a personal and professional level, what things do you think you had to give up to take care of your child with Dravet syndrome?
13. In number, how many hours a day do you dedicate to the care and attention of your child with Dravet syndrome that you would not otherwise dedicate to them?
14. How much time do you spend on yourself at home and doing activities related to self-care?
15. Do you feel support from the rest of the family members in caring for the person with Dravet Syndrome?
    - Not as much as I'd like.
    - They always help me with what I need.
    - They don't help me at all.
    - We don't have any other cohabitants at home.
16. How overloaded do you feel?
    - A lot.
    - Something.
    - Little.
    - Nothing.
17. If you are the primary caregiver, have you considered delegating some of your responsibilities to continue caring for the family member with Dravet syndrome? For example, hiring someone to clean or cook at home.
    - Yes, we have outside help.
    - Yes, we would like to, but we don't have the financial means to hire someone.
    - No.
18. If you have help caring for your family member with Dravet syndrome, do you feel bad about yourself for not being able to be there for as long as possible?
    - Yes.
    - No.
    - We don't have help.
19. How many days a week can you feel bad because of that?
    - None.
    - 1-2 days a week.
    - 3-4 days a week.
    - 5-6 days a week.
    - Every day.
20. Since you've been in this person's care, have you ever allowed yourself to have a bad day in their presence?
    - Often.
    - Sometimes.
    - Never.
21. If any, what aspects of your life have benefited from this situation of caring for a person with Dravet syndrome?

**Section 7**

- - 1. How many days a week do you usually dedicate time for leisure activities?
       - None.
       - 1-2 days a week.
       - 3-4 days a week.
       - 5-6 days a week.
       - Every day.
    2. How many of them do you do away from home alone or with friends or other family members you are not in charge of?
       - None of them.
       - Just a few very specific ones.
       - A lot of them.
       - All.
    3. In those leisure activities that you do at home, can you be attending to that activity 100%?
       - Yes, always.
       - Sometimes.
       - Not as much as I'd like.
       - I can almost never switch off 100%.
       - I never do leisure activities at home.
    4. Have social relationships (friends, co-workers, etc.) been modified as a result of caring for this person with Dravet syndrome?
       - I still have the same social relationships as before.
       - I only maintain a few social relationships (the closest ones).
       - I retain virtually none of the social relationships prior to Dravet syndrome.
    5. Have you had to look for more favourable environments to be able to be with the person with Dravet Syndrome?
       - Yes.
       - No.
    6. Do you think there are enough leisure environments adapted enough for you to be able to carry out an activity with your Dravet child with some safety? For example, inclusive parks or adapted cinemas with lower, brighter sound.
       - Yes.
    7. Do you feel more comfortable in an environment where you can share your experience with others who are in a similar situation?
       - Always.
       - Sometimes.
       - Rarely.
       - I feel equally comfortable in all settings regardless of whether the people I'm with are in a similar situation or not.
    8. Why do you think you can feel more comfortable with people who are in the same situation as you? If not, why do you think other people might feel more comfortable?
    9. Has this situation allowed you to create new social relationships?
       - Yes, quite a few.
       - Yes, some.
       - Yes, although very few.
       - I haven't created new social relationships.
    10. Do you think you've lost friendships because of this situation?
        - Yes, quite a few.
        - Yes, some.
        - Yes, although very few.
        - I'm not sorry I lost any friendships because of this situation.

**Section 8**.

1. Do you know of any public or private resources that can help you in your situation?
   - Yes.
   - No.
2. If yes, please indicate which one(s):
3. Does your family currently benefit from or have access to any of the following resources?
   - Social assistance services.
   - Psychological assistance services.
   - Rehabilitation services (Occupational Therapy, Physiotherapy, Speech Therapy...).
   - Financial aid.
   - Telephone support.
   - Personal Care Assistant.
   - Help with household chores.
   - Other:
   - I don't benefit from or have access to any resources.
   - No, none.
4. Which institution provides social assistance services?
   - Town hall or municipal services.
   - Provincial Council.
   - Social Services of the Autonomous Community.
   - Regional health system of the Autonomous Community.
   - Foundations and non-profit organizations.
   - Other:
   - None.
5. In case the person with Dravet syndrome is 18 years of age or older, what is their legal status?
   - Situation of extension of parental authority.
   - With a legal guardian.
   - Full legal capacity.
   - He is not yet of legal age.
   - Other:
6. How long did you wait until you started receiving any kind of public aid if you had applied for it?
7. Was it easy for you to access the services offered by the public system?
   - Yes.
   - No.
8. What is most important to you when applying for public aid?
   - Easy access to the resource.
   - Easy access to information and requirements.
   - May the resolution be beneficial to my family.
   - Other:
9. Do you think you receive the necessary help from public administrations in general?
   - Yes.
   - No.
10. What is the average monthly amount of money you spend on care, treatment, therapies and therapeutic items not covered by public administrations?
11. Regarding disability and dependency, did social or health services explain to you and was it clear to you how obtaining the degree of disability and dependency works, as well as the related aid available?
    - Yes, they explained it well to us and it was clear to us
    - Yes, but in a poor way and it wasn't clear to us at all
    - No
12. How long did it take you to get the degree of disability of the person with Dravet syndrome since you started the process?
13. How long did it take you to achieve the degree of dependency of the person with Dravet syndrome since you started the process?
14. Are you satisfied with the assessment of the degree of dependency and disability?
    - Yes.
    - No, and we have or are resorting
    - No, but we haven't appealed
